# A Vesicular Protein Important for Puberty

**DOI:** 10.1371/journal.pbio.1001953

**Published:** 2014-09-23

**Authors:** Caitlin Sedwick

**Affiliations:** Freelance Science Writer, San Diego, California, United States of America

The outward signs of puberty—for example, accelerated growth and the appearance of body hair—are no less dramatic than the accompanying internal changes, which include increased release of steroid hormones from the pituitary gland and the maturation of the sexual organs. All these changes are sparked by increased release of gonadotropin-releasing hormone (GnRH) from hypothalamic neurons. But in rare cases, such as in persons with a condition known as hypogonadotropic hypogonadism, the gonads fail to develop properly, and puberty may be delayed or absent altogether. This condition can stem from a variety of genetic mutations, the study of which has led to important insight into the processes that control puberty onset. In their paper published in this month's *PLOS Biology*, Brooke Tata, Lukas Huijbregts, Nicolas de Roux, and colleagues describe a new syndrome with a complex neurophysiological phenotype that includes hypogonadotropic hypogonadism, and the authors explore the genetic and molecular mechanisms that drive it.

**Figure 1:**
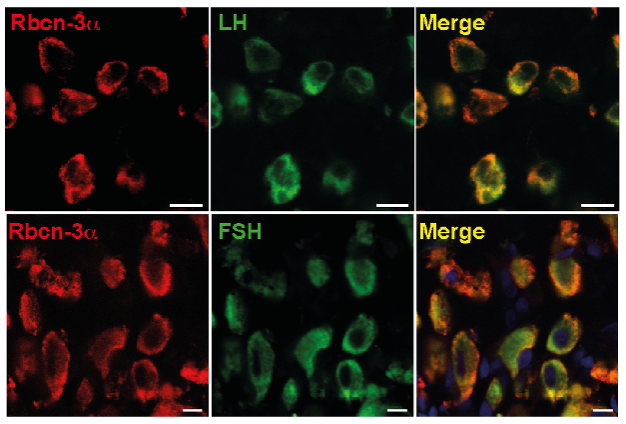
Rbcn-3α is a synaptic protein that is also expressed in luteinizing hormone (LH) and follicle-stimulating hormone (FSH) expressing cells in adult rat pituitary glands and needed for the central control of reproduction.

Tata et al. investigated a trio of brothers who share a previously undescribed disorder. The syndrome first manifested in early childhood with slowed growth and expanded with age to include a wide variety of deficits, including the development of progressive non-autoimmune diabetes mellitus, demyelinating polyneuropathy that caused difficulty in walking, and incomplete puberty. What could cause such disparate symptoms?

The syndrome affected neither the brothers' parents nor their two sisters, suggesting the condition might be caused by a recessive inherited trait. Subsequently, genomic analysis identified two candidate regions, each on different chromosomes, which could harbor the mutation. To identify the mutation, the researchers conducted high-throughput sequencing of the genes contained in these regions on samples collected from one of the patients and his mother. Comparison of the sequenced genes revealed that the patient indeed bore a mutation: an in-frame deletion of five codons in the gene *DMXL2*, which encodes a protein called rabconnectin-3α (Rbcn-3α). The patient was homozygous for the mutation, having inherited a mutated copy of the *DMXL2* gene from both his mother and his father. By contrast, his mother, father, and one sister were heterozygous for the mutation, such that each bore one normal and one mutated copy of the gene (the remaining sister had inherited only normal copies of the gene from both parents). This pattern was consistent with the syndrome's recessive mode of inheritance.

Tata et al. noticed that the brothers expressed significantly less *DMXL2* mRNA in blood lymphocytes than did their siblings or parents, leading the authors to hypothesize the syndrome could result from a deficiency in *DMXL2* expression. To test the proposed link between *DMXL2* expression and the observed phenotype, the authors analyzed the expression of the gene in rodents. Rbcn-3α is commonly found at neuronal synapses. Consistent with this, the researchers found that both *Dmxl2* mRNA and the Rbcn-3α protein were present in several brain regions, including the hypothalamus. Rbcn-3α was also expressed in gonadotropes (endocrine cells that release hormones involved in puberty) in the pituitary gland.

To directly test whether decreased *DMXL2* can cause a phenotype in mice similar to that in humans, the authors created transgenic mice with a truncated version of the gene. However, homozygous mutant mice died in utero, indicating that complete loss of the gene has severe consequences for development. Tata et al. therefore generated a new set of transgenic animals in which loss of *Dmxl2* was confined to neurons. In these animals, heterozygotes had only half the amount of *Dmxl2* mRNA in their hypothalamus as was present in controls and showed delayed puberty and decreased fertility. This stemmed from reduced levels of GnRH that, in turn, resulted from a loss of hypothalamic GnRH-releasing neurons that occurred for unknown reasons. Interestingly, the heterozygous mice did not display any signs of neuropathy, indicating that their *Dmxl2* levels were sufficient to stave off this effect.

As *DMXL2* loss was also associated with metabolic disorder in the three brothers, the authors examined whether *Dmxl2* might affect metabolic balance in mice. After a meal, a spike in glucose levels causes pancreatic beta cells to release insulin, which encourages glucose uptake by other cells of the body. Non-autoimmune diabetes mellitus, the condition suffered by the three brothers in this study, is frequently caused by failure of pancreatic beta cells to release insulin and results in the accumulation of blood glucose to toxic levels. Importantly, Tata et al. found that Rbcn-3α was present in pancreatic beta cells in mice, and that siRNA-mediated knockdown of *Dmxl2* expression in an insulin-secreting cell line strongly impaired insulin release.

These clues, taken together, show that decreased *DMXL2* levels could be responsible for both the incomplete puberty and the diabetes mellitus experienced by human patients carrying homozygous deletions in the gene. Additionally, examination of the mechanisms causing loss of GnRH neurons in *Dmxl2*-deficient animals could offer important new information on how the GnRH system is regulated and controlled during puberty onset.

**Tata B, Huijbregts L, Jacquier S, Csaba Z, Genin E, et al. (2014) Haploinsufficiency of** ***Dmxl2*** **, Encoding a Synaptic Protein, Causes Infertility Associated with a Loss of GnRH Neurons in Mouse.** doi:10.1371/journal.pbio.1001952

